# Human Parathyroid Hormone (1–34) accelerates skin wound healing through inducing cell migration via up-regulating the expression of Rac1

**DOI:** 10.1186/s13008-024-00111-3

**Published:** 2024-02-12

**Authors:** Qingpeng Sun, Liya Zhou, Zhiyong Yu, Jun Zhang, Chao Zhang, Honglin Pi

**Affiliations:** Department of Orthopaedic, Xiangyang Hospital of Traditional Chinese Medicine, No. 24, Changzheng Road, Fancheng District, Xiangyang, 441000 Hubei Province China

**Keywords:** Wound healing, Skin, Rac 1, Cell migration, hPTH(1–34)

## Abstract

Delayed wound healing is a public issue that imposes a significant burden on both society and the patients themselves. To date, although numerous methods have been developed to accelerate the speed of wound closure, the therapeutic effects are partially limited due to the complex procedures, high costs, potential side effects, and ethical concerns. While some studies have reported that the in-vivo application of Human Parathyroid Hormone (1–34) (hPTH(1–34)) promotes the wound-healing process, the definitive role and underlying mechanisms through which it regulates the behavior of fibroblasts and keratinocytes remains unclear. Herein, hPTH(1–34)’s role in cell migration is evaluated with a series of in-vitro and in-vivo studies, whereby hPTH(1–34)’s underlying mechanism in activating the two types of cells was detected. The in-vitro study revealed that hPTH(1–34) enhanced the migration of both fibroblasts and HaCaT cells. Ras-associated C3 botulinum toxin subunit 1 (Rac1), a classical member of the Rho family, was upregulated in hPTH(1–34)-treated fibroblasts and HaCaT cells. Further study by silencing the expression of Rac1 with siRNA reversed the hPTH(1–34)-enhanced cell migration, thus confirming that Rac1 was involved in hPTH(1–34)-induced cell behavior. In-vivo study on rat wound models confirmed the effects of hPTH(1–34) on fibroblasts and keratinocytes, with increased collagen deposition, fibroblasts accumulation, and Rac1 expression in the hPTH(1–34)-treated wounds. In summary, the present study demonstrated that hPTH(1–34) accelerated wound healing through enhancing the migration of cells through the up-regulation of Rac1 expression.

**Supplementary Information:**

The online version contains supplementary material available at 10.1186/s13008-024-00111-3.

## Introduction

The process of skin-wound healing is a carefully orchestrated series of events that involves three interrelated phases: inflammation, tissue regeneration, and remodeling [1–3]. Throughout the healing process, a variety of cellular and molecular components are involved and play crucial roles in the repair of damaged skin [3]. However, despite the sophisticated design of the wound-healing process, complicating factors such as infection, malnutrition, and immune disorders can disrupt this process and potentially lead to a chronic, non-healing wound. To date, delayed or non-healing wounds have become a global issue that is imposing a considerable burden on both society and the patients themselves. Prior research has reported that more than 40 million people globally suffer from chronic wounds, while nearly 2% of individuals in developing countries might experience a chronic wound during their lifetime [4]. Therefore, it is vital to develop appropriate treatment methods to accelerate the wound-healing process, aiming to prevent or minimize the formation of chronic wounds.

It is widely accepted that fibroblasts and keratinocytes play key roles in the skin-wound-healing process [2]. Following a wound, fibroblasts migrate to the wound area and transform into myofibroblasts expressing α-smooth muscle actin (α-SMA) to contract the wound [1, 2, 5] In addition, during the early stage of wound healing, the extracellular matrix secreted by fibroblasts and myofibroblasts constitutes an indispensable component of the granulation tissue in the wound area. Meanwhile, keratinocytes undergo epithelial–mesenchymal transition, transforming from a resting phenotype to a migratory phenotype and rapidly migrating toward the center of the wound in order to cover the newly formed granulation tissue [6, 7]. Given the crucial roles of these two cell types in wound healing, various agents have been developed to accelerate the healing process by regulating their behavior, with recombinant growth factors (e.g. TGF-β, PDGF, FGF, and EGF) being the most widely reported [8]. However, the use of such recombinant growth factors for wound therapy has not been widespread in clinical practice. The potential challenges such as a short half-life, complicated and unclear mechanisms, and over proliferation, as well as the risk of undesirable effects like the increased risk of malignant tumor, inevitably limit their application. Therefore, agents that are relatively safe and available are worthy of further research.

Parathyroid hormone (PTH), a physiological hormone synthesized from parathyroid, plays a vital role in modulating the balance of calcium and phosphorus. As a truncated PTH peptide, hPTH(1–34) (teriparatide) is an effective agent in osteogenesis when being intermittently injected, thus is widely employed in the treatment of osteoporosis [9–11]. Additionally, it also exerts effects on angiogenesis and extracellular matrix (ECM) deposition [4, 12, 13]. However, the definitive role of hPTH(1–34) in skin-wound healing and the underlying mechanisms remain unclear, although a few studies reported that PTH or PTH analogs (PTHrP2 and MY-1) led to a quicker wound closure [4, 14–16]. To date, although the in-vivo application of PTH has been under-reported, there is a paucity of detailed experiments that demonstrate the definitive effects of PTH on fibroblasts and keratinocytes. With regard to the underlying mechanisms, while previous research suggested that PI3K/AKT, TGF-β and ERK signaling might have an important function in the PTH-induced wound-healing process [4, 14], the other mechanisms that mediate PTH’s role in wound repair require further exploration.

Rac1, a member of the Rho subfamily of small GTPases, is closely related to a variety of cell activities that include cytoskeleton reconstruction, cell migration, and proliferation, as well as cell survival [2, 17]. The expression of Rac1 is commonly observed in malignant tumor tissues, while the abnormal expression has been significantly associated with tumor growth, metastasis, and drug resistance [18, 19]. Besides malignant tumor fields, Rac1’s role in skin-wound healing was also reported in a few studies [20]. Previous studies revealed that the deletion or inhibition of Rac1 in skin-related cells led to a delayed wound-healing process [17], while inducement of the expression or activation of Rac1 with TGF-β1 accelerated the coverage of skin defects [2]. However, besides the indispensable role of Rac1 in wound healing, whether hPTH(1–34) accelerates skin-wound healing through regulating the expression of Rac1 remains unclear. This study investigated the role of hPTH(1–34) in skin-wound repair with in-vitro and in-vivo studies, as well as the relationship between the hPTH(1–34)-induced cell characteristics and the expression of Rac1.

## Results

### hPTH(1–34) promoted fibroblasts and HaCaT cells migration

**Fig. 1:**
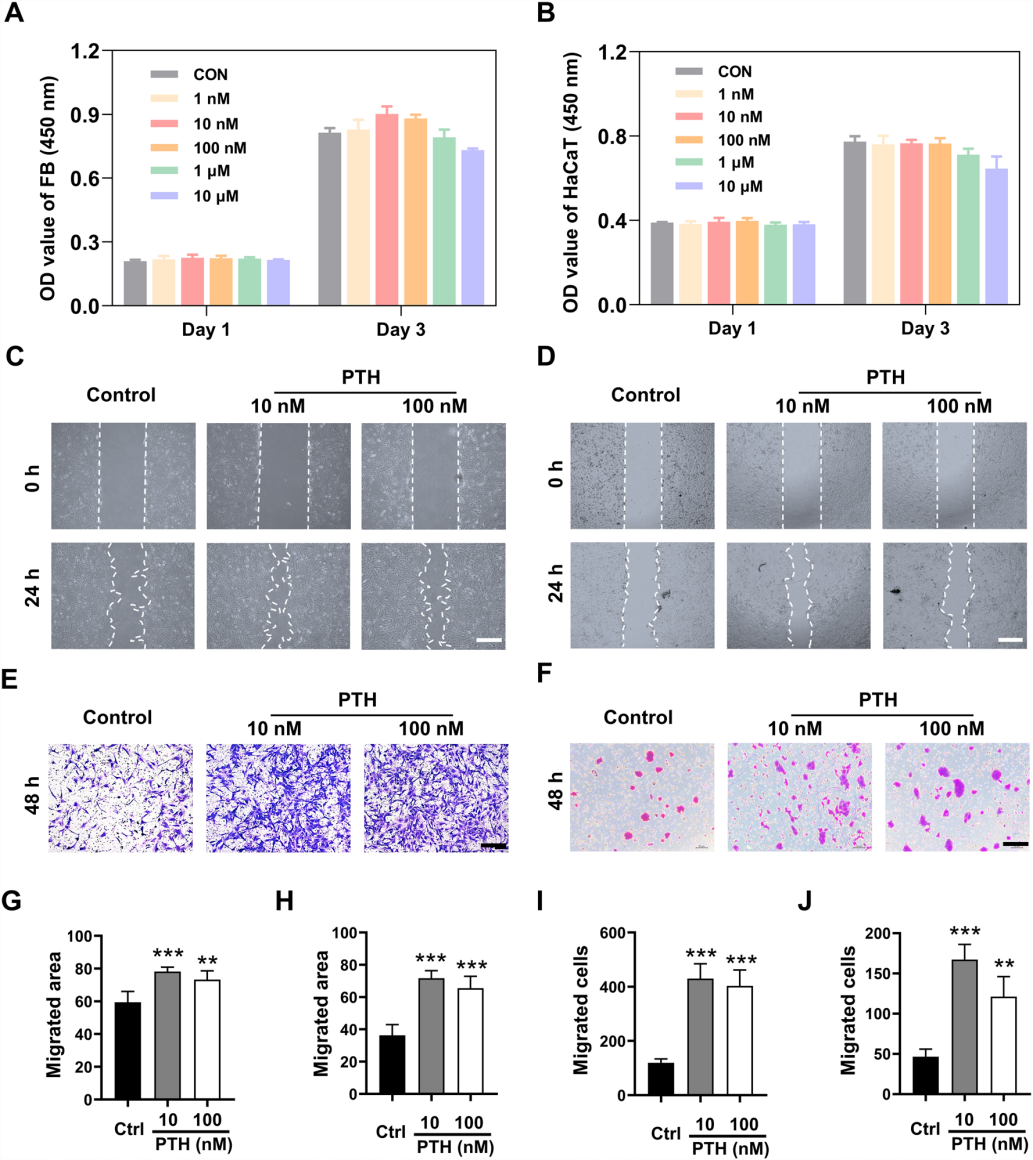
hPTH(1–34) treatment enhanced the motility of fibroblasts and HaCaT cells. **A** and **B** Cell viability was measured by CCK8 kit after fibroblasts and HaCaT cells were subjected to the graded concentrations of hPTH(1–34) (0–1 μM) for 24 h and 72 h. **C** and **D** Representative images of wound scratching assays on fibroblasts and HaCaT cells. Images were captured at 0 h and 24 h after incubating with / without hPTH(1–34). **E** and **F** Representative images of Transwell assays on fibroblasts and HaCaT cells after incubating with / without hPTH(1–34) for 48 h. **G** and **H** Quantification of the migrated areas in hPTH(1–34)-treated fibroblasts (**G**) and HaCaT cells (**H**) after subjected to hPTH(1–34) or PBS for 24 h. **I** and **J** Quantification of the migrated fibroblasts (**I**) and HaCaT cells (**J**) after subjected to hPTH(1–34) or PBS for 48 h. The data represent mean ± SD. *p < 0.05; **p < 0.01; ***p < 0.001 vs Control. Scale bar: 50 μm in (**C**–**F**). *Ctrl* Control

To examine the suitable concentrations and the potential effects of hPTH(1–34) on the cell culture, primary fibroblasts and HaCaT cells were employed for a series of assays. In CCK-8 assay, two kinds of cells were seeded in 96-well plates and treated with graded concentrations of hPTH(1–34) (1 nM to 10 μM) for 24 h or 72 h. Then, the cell viabilities were measured using a CCK-8 kit. The results of the CCK-8 assay indicated that, although there was no significant effect on cell proliferation, lower concentrations of hPTH(1–34) (10 nM and 100 nM) had the better effect on cell viability. In contrast, higher hPTH(1–34) concentrations (1 μM and 10 μM) led to an inhibitory effect on cell viability (Fig. 1A, B). These optimum concentrations of hPTH(1–34) on fibroblasts and HaCaT cells were consistent to the concentrations of hPTH(1–34) utilized in the other cells.

Since the cells in the 10 nM and 100 nM hPTH(1–34) groups showed the highest cell viability, for convenience, the fibroblasts and HaCaT cells were next treated with these concentrations and their role in cell migration evaluated. Cell scratching assay and Transwell assay were first utilized to evaluate whether hPTH(1–34) treatment regulated cell motility. In wound-scratching assay, the results exhibited that 24 h of hPTH(1–34) treatment led to narrower wound areas, which indicated that suitable concentrations of hPTH(1–34) (especially at 10 nM) enhanced the motility of fibroblasts and HaCaT cells (Fig. 1C, D, G, and H). These results were subsequently confirmed by the Transwell assays, in which hPTH(1–34) management drove more cells across the 8 µm member (Fig. 1E, F, I,and J). In summary, the findings confirmed hPTH(1–34)’s ability to promote fibroblasts and HaCaT cell migration.

### In-vitro application of hPTH(1–34) induced the Rac1-related cytoskeleton re-construction and lamellipodia formation

**Fig. 2:**
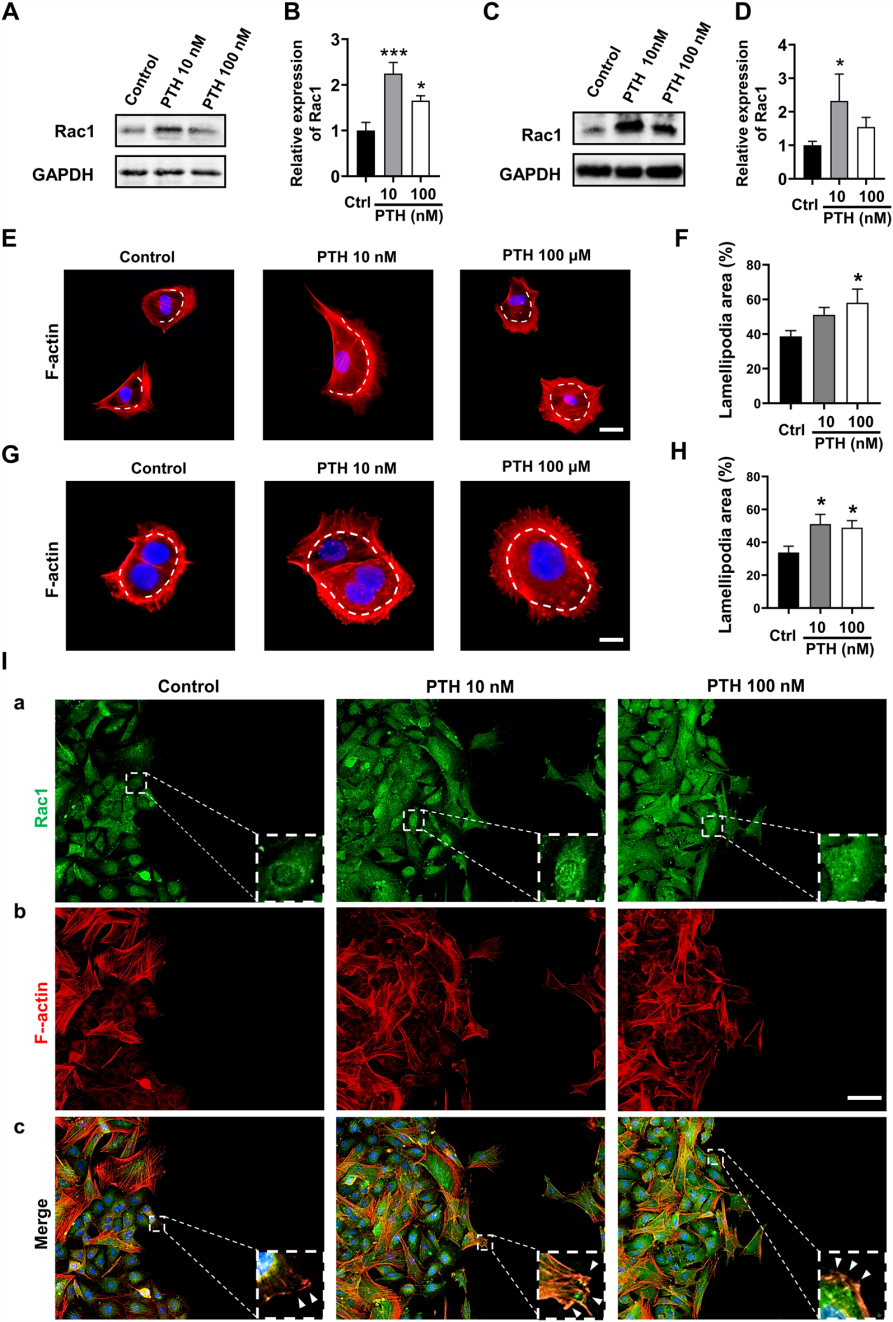
hPTH(1–34) treatment induced the Rac1-related cytoskeleton re-construction and lamellipodia formation. **A**–**D** The expression of Rac1 in fibroblasts (**A**) and HaCaT cells (**C**) were evaluated by western blot analysis after treated with or without hPTH(1–34). The intensity of each band was measured and normalized to GAPDH then calculated as the ratio of the controls (**B** and **D**). **E**–**H** Cell scattering assay on fibroblasts and HaCaT cells were carried out using rhodamine-phalloidin staining after incubating with different concentrations of hPTH(1–34) for 12 h (**E** and **G**). The ratio of lamellipodia areas versus total cell areas in fibroblasts (**F**) and HaCaT cells (**H**) were measured and quantified. **I** Immunofluorescent staining of Rac1 and F-actin were performed on fibroblasts which were subjected to different treatments for 12 h. Green, Rac1; Red, F-actin; Blue, DAPI. The data represent mean ± SD. *p < 0.05; **p < 0.01; ***p < 0.001 vs Control. Scale bar: 25 μm in (**E**), 10 μm in (**G**), 50 μm in (**I**). *Ctrl* Control

As Rac1 is a crucial molecule in regulating cell proliferation, migration, cytoskeleton reconstruction, and lamellipodia formation, the next evaluation considered whether the expression levels of Rac1 were upregulated though post-hPTH incubation. The results of western blot analysis revealed that the expression levels of Rac1 were improved in both the fibroblasts and HaCaT cells after co-culture with hPTH(1–34) for 24 h, indicating that Rac1 was one of the signaling regulated by hPTH(1–34) (Fig. 2A–D).

Since cytoskeleton reconstruction and lamellipodia formation driven by Rac1 is an indispensable process for cell motility acquisition, the lamellipodia areas and cytoskeleton reconstruction of cells were firstly measured and compared between the control and hPTH(1–34)-treated groups. The results of cell scattering assay revealed that fibroblasts and HaCaT cells in the hPTH(1–34) groups exhibited larger cell sizes compared to those in the control group (Fig. 2E–H). This phenomenon was related to cells expansion induced by the increased activities of the cytoskeleton reconstruction. In terms of the lamellipodia formation, the fibroblasts and HaCaT cells treated by hPTH(1–34) formed larger areas of lamellipodia in the their periphery (Fig. 2E–H), conforming the positive role of Rac1 in hPTH(1–34)-induced cell migration.

To evaluate whether hPTH(1–34) regulates the expression levels and distribution of Rac1 in cells, immunofluorescence staining of Rac1 and F-actin was carried out on migrating fibroblasts after being subjected to different treatments for 12 h. As shown in Fig. 2E a, hPTH(1–34) treatments notably improved the expression levels of Rac1 in the fibroblasts and drove Rac1 re-distribution throughout the fibroblasts. Recent study reported that the accumulation of Rac1 in nuclei is closely related to the capacity of cell migration and invasion [21]. Similarly, the present study confirmed this result, with significant Rac1 protein accumulation in the nucleus following the hPTH(1–34) treatment (Fig. 2I a). Moreover, hPTH(1–34) treatment also induced more active cytoskeleton re-construction, thereby resulting in lamellipodia formation in more fibroblasts (Fig. 2I b), which was consistent with the results of the cell scattering assay. Then, the relationship between the Rac1 distribution and F-actin reconstruction was evaluated. As shown in Fig. 2I c, the immunofluorescence co-location analysis of Rac1 and F-actin revealed that hPTH(1–34) treatment resulted in a sizeable accumulation of Rac1 along the lamellipodial edge (the leading edge of migration) where F-actin also localizes, which confirmed the close relationship between Rac1 and cytoskeleton reconstruction, as reported in the literature [22].

### Silencing of Rac1 inhibited the hPTH(1–34) induced migration ability in fibroblasts and HaCaT cells

**Fig. 3:**
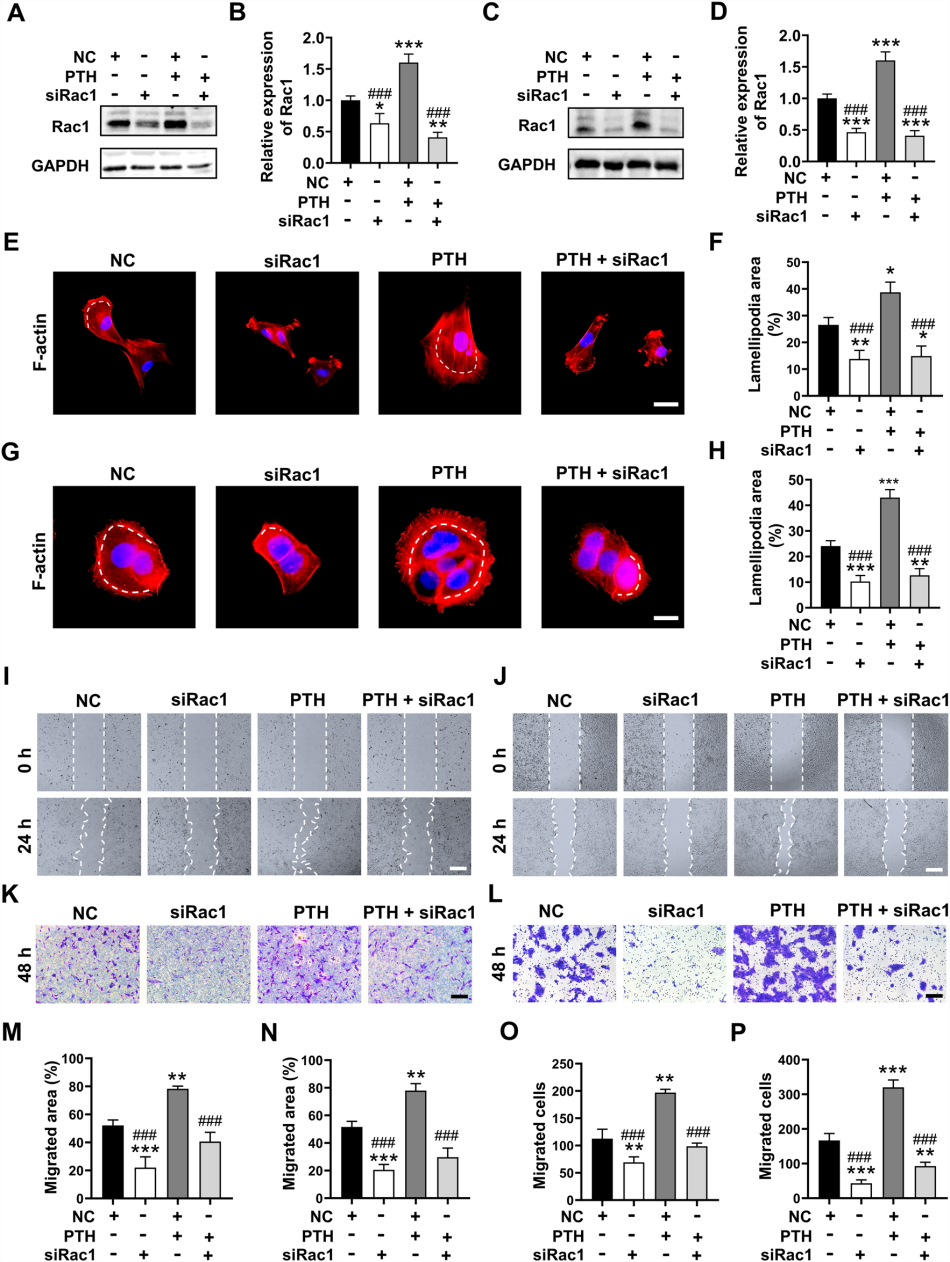
Silencing the Rac1 reversed the hPTH(1–34)-migration of fibroblasts and HaCaT cells. **A**–**D** The expression of Rac1 in siRNA-transfected fibroblasts (**A**) and HaCaT cells (**C**) were evaluated by western blot analysis after treated with or without hPTH. The intensity of each band was measured and normalized to GAPDH then calculated as the ratio of the controls (**B** and **D**). **E**–**H** siRNA-transfected fibroblasts (**E**) and HaCaT cells (**G**) were incubated with or without hPTH(1–34) for 12 h and the lamellipodia in two types of cells were stained with rhodamine-phalloidin. The ratio of lamellipodia areas versus total cell areas was measured in fibroblasts (**F**) and HaCaT cells (**H**). **I** and **J** Representative images of wound scratching assays on siRNA-transfected fibroblasts (**I**) and HaCaT cells (**J**) after incubating with or without hPTH(1–34). Images were captured at 0 h and 24 h. **K** and **L** Representative images of siRNA-transfected Transwell assay on fibroblasts (**K**) and HaCaT cells (**L**) after incubation with / without hPTH(1–34) for 48 h. **M** and **N** Quantification and analysis of the migrated areas in hPTH(1–34)-treated fibroblasts (**M**) and HaCaT cells (**N**) using imageJ software. **O** and **P** Quantification of the migrated fibroblasts (**O**) and HaCaT cells (**P**) after subjected to hPTH(1–34) or PBS for 48 h. The data represent mean ± SD. *p < 0.05; **p < 0.01; ***p < 0.001 vs control group. #p < 0.05; ##p < 0.01; ###p < 0.001 vs hPTH(1–34) group. Scale bar: 25 μm in (**E**), 10 μm in (**G**), 50 μm in (**I**–**L**). *NC* Negative control

To confirm whether hPTH(1–34) induced cell proliferation and migration through activating Rac1 signaling, specific small interference RNA targeting Rac1 (siRac1) and the negative control were subsequently employed. Following siRNA transfection, the expression levels of Rac1 in two types of cells were first evaluated. The qPCR analysis results demonstrated a significant reduction in Rac1 gene expression following siRac1 transfection (Additional file 1: Figure S1A, B), indicating the successful delivery of siRac1 into both fibroblasts and HaCaT cells. In addition, western blot analysis confirmed that increased Rac1 expression post- hPTH(1–34) treatment was successfully inhibited after siRac1 transfection (Fig. 3A–D).

Then, the cell migration capacity of two types of cells were evaluated. Since cytoskeleton reconstruction and lamellipodia formation are closely related to the Rac1 activation, the lamellipodia areas and cytoskeleton reconstruction were evaluated with the cell scattering assays. The results showed that the silencing of Rac1 resulted in the smaller cell sizes and impaired lamellipodia formation in siRac1-transfected groups (Fig. 3E–H). These findings suggested that the inhibited the expression of Rac1 partially reversed of the hPTH(1–34)-induced motility of two cell types. Then, the migration capacity of the fibroblasts and HaCaT cells in different groups was measured using wound-scratching and Transwell assays. Similarly, the hPTH(1–34)-enhanced cell migration ability was remarkedly inhibited after Rac1 was silenced, with larger wound areas remaining in the scratching assays and less migrated cell numbers in the Transwell assays (Fig. 3I–P).

Collectively, it was concluded that hPTH(1–34) induced in-vitro fibroblasts and HaCaT cell proliferation and migration through the upregulating expression of Rac1.

### Local injection of hPTH(1–34) accelerated skin wound healing in rat models

**Fig. 4:**
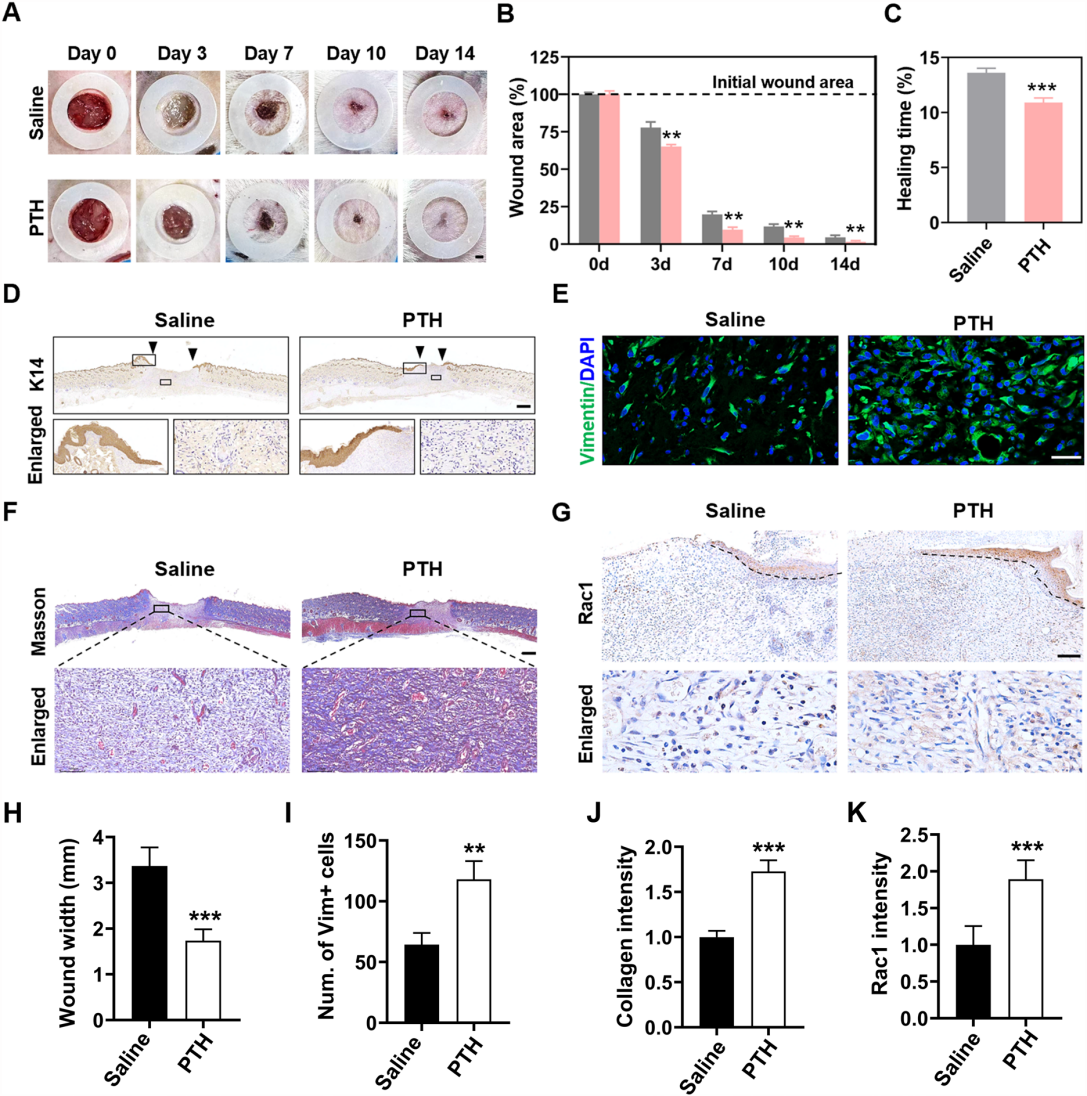
Injection of hPTH(1–34) accelerated skin wound healing in rat models. **A** and **B** Full-thickness skin wounds (diameter, 15 mm) were generated on the back of rats. hPTH(1–34) or saline was locally injected around the wounds, followed by covering the wounds with sterilized dressing. Wound images were captured at day 0, 3, 7, 10, and 14 (**A**), and wound area at each timepoint were measured and calculated (**B**). **C** Quantitation of the wound healing time in the hPTH(1–34) and saline groups. **D** Immunohistochemical staining of K14 was carried out on wound samples harvested at day 7 post-surgery. **E** Immunofluorescence staining of Vimentin were performed on wound samples at day 7 post-surgery. **F** Masson’s trichrome staining was carried out on wound samples harvested at day 7 post-surgery. **G** Immunohistochemical staining of Rac1 was carried out on wound samples. **H** Measurement of the wound widths in two groups after immunohistochemical staining of K14. **I** Measurement of the number of Vimentin positive cells after immunofluorescence staining of Vimentin. **J** Measurement of the collagen intensity in two groups after Masson’s trichrome staining was carried out. **K** Measurement of the intensity of Rac1 expression in two groups after immunohistochemical staining of Rac1 was carried out. The data represent mean ± SD. *p < 0.05; **p < 0.01; ***p < 0.001 vs saline group. Scale bar: 3 mm in (**A**); 1 mm in (**D** and **F**), 50 μm in (**E**). *K14* cytokeratin 14

To investigate the effects of hPTH(1–34) on skin-wound healing, hPTH(1–34) or saline was locally injected into cutaneous wounds on the back of rat models. Analysis of the wound closure rate between the two groups revealed that hPTH(1–34) significantly accelerated the wound-healing process when compared to the wound areas in the saline group (Fig. 4A). The wound areas in the hPTH(1–34) group sharply reduced from day 3 to day 7, and reached near-complete closure on day 10, much quicker than those wounds treated with saline (Fig. 4B). Moreover, when comparing the wound-healing duration, it took approximately 11 days for the hPTH(1–34)-treated wounds to achieve complete healing, which was much shorter than a mean healing time of approximately 14 days in the saline group (Fig. 4C).

Histological analysis was then employed to compare the histological difference between the two groups. The role of hPTH(1–34) on the wound re-epithelialization was first evaluated, whereby the IHC staining of Cytokeratin 14 (K14), a special marker of migrating keratinocytes (basal keratinocytes), revealed that 7 days of hPTH(1–34) administration resulted in longer epithelial tongues and narrower wound gaps, indicating that hPTH(1–34) promoted the process of re-epithelialization (Fig. 4D, H). Moreover, the local application of hPTH(1–34) induced thicker granulation tissues and an increased number of cells in the wound area, which might be explained by the pro-migration effects of hPTH(1–34) on fibroblasts (Fig. 4D, H). To confirm the role of hPTH(1–34) in cell proliferation and migration, the IF staining of Vimentin (a special marker of fibroblasts) was carried out in the wound areas. As shown in Fig. 4E, the local application of hPTH(1–34) induced more fibroblasts accumulated in the wound area (Fig. 4E, I), which confirmed the positive role of hPTH(1–34) in cell migration.

Since deposited collagen constitutes the main component of granulation tissues, the collagen deposition in the wound areas was then evaluated. Masson’s trichrome staining showed denser collagen fibers deposited in the hPTH(1–34)-treated wounds, suggesting that hPTH(1–34) promoted granulation tissue formation through inducing collagen secretion and deposition (Fig. 4F, J). Finally, the expression levels of Rac1 were compared between the two groups. As shown in Fig. 4G, the IHC staining of Rac1 revealed a higher expression of Rac1 in the hPTH(1–34)-treated wounds (Fig. 4G, K), demonstrating that hPTH(1–34) accelerated wound healing via the up-regulated expression of Rac1.

## Discussion

Delayed wound healing represents a global concern that imposes significant burdens on both society and the patients. Accelerating the wound-healing process is therefore of significant importance in terms of reducing or preventing the transformation of an acute wound into a chronic one. Currently, although various methods for wound administration have been developed, their application remains restricted by challenges involving complex treatment processes, increased healthcare costs, and ethical concerns. PTH as a physiological hormone has been recognized as an effective agent in anti-osteoporosis, angiogenesis, or bone-repair therapy, although its role in skin-wound healing is not widely reported, which is likely attributable to the potential hyper-activation of multiple organ functions when applied systemically. In order to avoid the potential shortcomings of systemic application, in the present study the biological function of hPTH(1–34) in wound healing was explored with a local injection. This study on in-vivo wound samples and in-vitro cells revealed that the administration of hPTH(1–34) accelerated the skin-wound-healing process by inducing collagen deposition, while promoting cell proliferation and migration. Mechanistically, the present research on two types of cells revealed Rac1 to be one of the key molecules that mediated the hPTH(1–34)-induced cell characteristics.

It is widely acknowledged that enhanced cell proliferation, migration, and collagen secretion contribute to the wound-healing process. PTH’s role in the wound-healing process has been investigated in a few studies. For example, Recent study of Stao et al. reported that TIP39 as a member of parathyroid hormone ligand family was widely expressed in the skin tissues and played a positive role on collagen deposition, decorin and fibronectin secretion via binding with PTHR2 [15]. Yao et al. reported a PTH-loaded phase-transition microneedle (PTMN) patch markedly enhanced wound healing process attributing to PTH induced re-epithelialization, collagen deposition and cells proliferation through TGF-β/Smad3/mTOR pathways [4]. However, neither of those two studies included in-vitro assays to evaluate the direct role of PTH in the motility of fibroblasts or keratinocytes. In another study, *Shen *et al. reported a newly designed PTH derivative, PTHrP-2, could activate the β-catenin and Akt/Erk1/2 signaling to accelerated wound healing process through inducing the migration of HUVECs and fibroblasts [14]. However, PTHrP-2 was pointed out to be ineffective in the migration of keratinocytes, which might be attributed to the differences in the structure of PTHrP-2 and original PTH. Therefore, to date the definite role of PTH in the migration of fibroblasts and keratinocytes remains obscure. To detect the PTH’s role on fibroblasts and keratinocytes, we treated two types of cells with hPTH(1–34) and evaluated the motility of cells through a serial of assays. Our study revealed that hPTH(1–34) administration significantly promoted the migration of cells, which undoubtedly increased the understanding of PTH in wound repair process.

Rac1 is a highly versatile member of the Rho family of GTPases, which is known for its critical role in regulating various cellular processes, and especially for regulating cytoskeleton dynamics, the cell–cell junction, and cell motility. In migrating cells, activated Rac1 drives the cytoskeleton polymerization to form lamellipodia at the leading edge through activating the PAK and WAVE complex in the downstream, thereby providing the driving force for cell movements [23, 24]. The central role of Rac1 in skin-wound healing has also been demonstrated in a number of studies. For instance, James et al. discovered that the mechanical stimulation of skin with ultrasound accelerated skin-wound healing in elderly or diabetic mice, through promoting fibroblast migration via activating the calcium/CamKinaseII/Tiam1/Rac1 pathway signaling [25]; Rogerio et al. reported that the conditional epidermal knockout of the Rac1 gene led to delayed cutaneous wound healing [17]; and Tang et al. revealed that Rac1 is an indispensable molecule that mediates the TGF-β-induced keratinocytes motility. The inhibition of Rac1 expression in epidermis or HaCaT cells with microRNA-200b led to damaged cell motility and eventually resulted in a substantially delayed wound-healing process [2]. PTH and its analogs have been recognized as effective pro-migration agents in various types of cells which at least included HUVECs [26], BMSCs, fibroblasts [14]. However, the relationship of PTH and the cell motility-related signaling, the Rac1 signaling, remains lack of studied. Herein, we firstly evaluated the Rac1 activation in both fibroblasts and HaCaT cells following hPTH(1–34) incubation. Cell cytoskeleton staining revealed that hPTH(1–34) incubation led to increased Rac1 expression and more active cytoskeleton reconstruction, while the inhibition of Rac1 expression in fibroblasts and HaCaT cells reversed the impact of hPTH(1–34) on cell migration. Consistently, cell scattering assays demonstrated that cells in hPTH(1–34)-treated groups exhibited larger sizes and lamellipodia areas compared to those in the control or siRac1 groups, suggesting the speeds of Rac1-driven cytoskeleton reconstruction were positively regulated by hPTH(1–34). Consequently, the results of this study indicate that Rac1 is involved in hPTH(1–34)-induced fibroblasts and HaCaT cell migration.

## Conclusion

In summary, our study revealed that hPTH(1–34) promotes wound healing by promoting the migration of fibroblasts and keratinocytes. In-vitro studies on primary fibroblasts and HaCaT cells revealed hPTH(1–34) enhanced cells migration by activating the Rac1 signaling. Therefore, it was concluded that hPTH(1–34) is a potential therapeutic agent for wound healing by regulating fibroblasts and keratinocytes motility.

## Methods and materials

### Cell culture

For primary fibroblast isolation, the skin tissues were obtained from 1-day-old Sprague–Dawley rat neonates and digested with 0.1% dispase (cat #: 11097113001, Sigma-Aldrich, St. Louis, MO, USA) overnight at 4 °C to separate the dermis from the epidermis. The dermis was cut into small pieces and digested with 0.25% trypsin (cat #: 25200072, Gibco, Rockville, MD, USA) and 0.2% type IV collagenase (cat #: C917427, Mecklin, Shanghai, China) at 37 °C for 15 min. Then, Dulbecco’s modified Eagle’s medium (DMEM; cat #: C11965500BT, Gibco)) containing 10% fetal bovine serum ([FBS]; cat #: 10270106, Gibco) and 1% penicillin and streptomycin (cat #: 15140122, Gibco) was added to inactivate the trypsin and type IV collagenase. The digested solution was collected and filtered with a 70 μm cell strainer (cat #: 352350, Falcon, USA) and centrifuged at 1000 rpm for 5 min to collect the isolated fibroblasts. The precipitated cells were re-suspended with complete medium, seeded in 100 mm dishes, and cultured in a cell culture condition of 5% CO_2_ and 95% humidity at 37 °C. The culture medium was changed every 2 days and the fibroblasts were passaged after reaching 80–90% confluence. The fibroblasts at passage 3–5 were utilized for the experiments.

Human immortalized keratinocyte cell line (HaCaT) was purchased from the Cell Bank of the Chinese Academy of Sciences (Shanghai, China). The cells were cultured with DMEM containing 10% FBS (Gibco) and 1% penicillin and streptomycin (Gibco) in a cell culture condition of 5% CO_2_ and 95% humidity at 37 °C. The culture medium was changed every 2 days and the HaCaT cells were passaged after reaching 80–90% confluence.

### CCK-8 assay

Fibroblasts and HaCaT cells were seeded in 96-well plates with a density of 2 × 10^3^ cells per well. After adhesion, the mediums were replaced with complete mediums containing graded concentrations ranging from 1 nM to 10 μM for 24 h or 72 h incubation. Then, the mediums were removed and 100 μl fresh DMEM (Gibco) containing 10 μl CCK-8 solution (cat #: CK04, Dojindo, Kumamoto, Japan) was added and incubated at 37 ℃ for 2 h. The optical density value at the 450 nm wavelength was measured via a SpectraMax i3x multi-mode microplate reader (Molecular Devices Co., Ltd, San Jose, CA, USA).

### Scratch wound-healing assay

Fibroblasts and HaCaT cells (4.5 × 10^4^ cells per well) were seeded in a 12-well plate until reaching 100% confluency. Then, the medium was removed and scratch wounds were created using sterile yellow pipette tips. The cell fragments were removed with a wash of phosphate buffered saline ([PBS]; cat #: C10010500BT, Gibco) and the DMEM containing hPTH(1–34) (0 nM, 10 nM, and 100 nM) was added into related wells. The pictures from different groups were captured at the 0 h and 24 h timepoints. The areas of the wounds were measured by ImageJ.

### Transwell assay

For the Transwell assay, the fibroblasts and HaCaT cells were re-suspended in a serum-free medium and adjusted to a concentration of 5 × 10^5^ cells per ml. Thereafter, 100 ul of suspended HaCaT cells was loaded into the upper chamber of a 24-well, 8 um pore-size Transwell plate (cat #: 3422, Corning, NY, USA). The culture medium containing 2% FBS (Gibco) with different mediums was added into the lower chamber. After 48 h of incubation, the cells in the upper chamber were fixed with 4% paraformaldehyde (cat #: DF0135, Leagene, Beijing, China) for 15 min and the unmigrated cells in the upper chamber were swabbed using cotton swabs. The migrated cells were stained with 0.1% (w/v) crystal violet (cat #: DA0061, Leagene) for 10 min and the images were photographed by IX73 inverted phase-contrast microscope (Olympus, Tokyo, Japan). The number of cells in each group was measured by ImageJ.

### Cytoskeleton (F-actin) staining

For cytoskeleton and lamellipodia staining, the fibroblasts and HaCaT cells were seeded in 24-well glass coverslips at a density of 1 × 10^4^ per well. After adhesion, the complete medium was exchanged with a DMEM medium (featuring 2% FBS) with or without hPTH(1–34) and incubated for 12 h. Then, the fibroblasts and HaCaT cells in each group were fixed with 4% paraformaldehyde for 15 min, washed with PBS (Gibco) three times and stained with rhodamine-phalloidin (1:1000; cat #: ab235138, Abcam, Cambridge, UK) for 30 min. Finally, 4',6-diamidino-2-phenylindole ([DAPI]; cat #: P0131, Beyotime) was employed to stain the nucleus for 10 min. The cell morphology and lamellipodia were observed by BX63 microscope (Olympus).

### Immunofluorescence staining of fibroblasts

For immunofluorescence staining, the fibroblasts were first fixed with 4% paraformaldehyde for 15 min. Then, the fibroblasts were permeabilized with a 0.5% (v/v) Triton X-100 (cat #: T8200, Solarbio, Beijing, China)) in PBS for 15 min, blocked in 5% bovine serum albumin (cat #: SW3015, BSA, Solarbio) for 1 h and incubated in primary antibodies overnight at 4 ℃. Subsequently, the cells were incubated in Alexa Fluor 488 fluorescence-conjugated secondary antibodies (1:1000; cat #: 150077, Abcam) at room temperature for 2 h. Finally, DAPI staining solution (Beyotime) was utilized to stain the nuclei of the cells for 5 min. Images were collected by BX63 microscope (Olympus). The primary antibodies used in immunofluorescence staining included anti-Rac1 (1:250; cat #: 24072–1-AP, Proteintech, Beijing, China).

### siRNA transfection

Small interference RNA targeting Rac1 and negative control was designed and synthesized by Qingke Co., Ltd. (Beijing, China). siRNA transfection was carried out using Lipo3000 Transfection Reagent (cat #: L3000015, Invitrogen; Carlsbad, CA, USA) according to the manufacturer’s instructions, when the primary fibroblasts and HaCaT cells had reached 80% confluence.

### Quantitative real-time PCR (qRT-PCR) analysis

Total RNA was extracted with an RNA Purification Kit (cat #: RC101-01, Vazyme, Nanjing, China) and reverse transcription was performed with a cDNA Reverse Transcription Kit (cat #: R223-01, Vazyme, Nanjing, China). qRT-PCR was performed using SYBR Green qPCR Master Mix ((cat #: Q121-02, Vazyme, Nanjing, China). Relative gene expression was calculated using the 2^−ΔΔCT^ method, and GAPDH was used as a reference for normalization. The qPCR primers were listed in Additional file 2: Table S1.

### Western blotting analysis

Total protein of fibroblasts and HaCaT cells were extracted using the RIPA lysis buffer (cat #: P0013B, Beyotime) with protease inhibitor and protein phosphatase inhibitor (cat #: P1045, Beyotime) on the ice. 30 μg of protein were subjected to 10% SDS-PAGE (cat #: P0015, Beyotime) and blotted onto PVDF membranes (cat #: IPVH00010, Millipore, St. Louis, MO, USA). After blocked with QuickBlock™ Blocking Buffer for Western Blot (cat. no. P0239, Beyotime), the membranes were incubated with primary antibodies against Rac1 (1:1000 dilution, Proteintech) and GAPDH (1:1000 dilution, cat #: 60004-1-Ig, Proteintech) at 4 °C overnight. Then, the membranes were washed with TBST and incubated in HRP-conjugated secondary antibodies (1:3000 dilution, cat #: #S0001 and #S0002, Affinity, Beijing, China) at room temperature for 2 h. The proteins in each group were visualized by Gelview 6000 Pro (BLT Co., Ltd, Guangzhou, China) and the bands were analyzed with Image J software.

### In-vivo wound model

Male Sprague–Dawley rats (SPF, 250 ± 30 g) were purchased from the Animal Center of Southern Medical University and kept at 20–22 °C under a 12:12 light/dark cycle (animal experiments approved by the Animal Ethics Committee of Xiangyang Hospital of Traditional Chinese Medicine: Grant No. XYZY2023017). After anesthetizing by the inhalation of isoflurane (cat #: R5100, RWD. Shenzhen, China), two symmetric full-thickness skin wounds (diameter: 15 mm) were created on the back of the rats with the help of a puncher and scissor. The rats were randomly grouped and the wound received an equal volume of saline or hPTH(1–34) injection (0.1 μg/100 μl) on a daily basis. After treatment, the wounds were covered with Tegaderm^™^ films (3 M, St. Paul, MN, USA) and protected with gauze and bandages. The dorsal wounds were photographed on days 0, 3, 7, 10, and 14 post-surgery, with the healing effects evaluated with ImageJ. Rats from each group were humanely sacrificed on day 7 post-surgery, with the wound samples harvested and fixed in 4% paraformaldehyde.

### Histological, immunohistochemical, and immunofluorescence staining

Wound samples were harvested at certain timepoints and fixed in 4% paraformaldehyde for 24 h. Then, the samples were dehydrated through graded ethanol of increasing concentration and embedded in paraffin. For the immunohistochemical staining, 4-μm-thick sections were firstly deparaffinized in xylene twice and rehydrated in a graded series of ethanol and PBS. Then, dewaxed sections underwent antigen retrieval in a microwave oven for 20 min and were then immersed in 3% H_2_O_2_ for 15 min to block endogenous peroxidase activity. Next, the samples were incubated in goat serum for 30 min at room temperature to block the non-specific antigen, followed by incubating in primary antibodies against K14 (1:200; cat #: sc-53253, Santa Cruz, Cincinnati, OH, USA) and Rac1 (1:250; Proteintech) overnight at 4 ℃. Finally, the immune reactivities of the sections were determined using the HRP‐streptavidin detection system (cat #: ZLI-9017, ZSGB-bio, Beijing, China). Images from the above-mentioned sections were acquired with the help of a digital pathological section scanner (Olympus).

For immunofluorescence staining, 4-μm-thick sections received antigen retrieval for 20 min in a microwave oven and were then permeabilized with 0.5% Triton X-100 (Solarbio) for 15 min. After blocking with goat serum (Solarbio), primary antibodies against Vimentin (1:200; cat #: sc-6260, Santa Cruz) were loaded and kept at 4 ℃ overnight. Then, the primary antibodies were removed and Alexa Fluor 488- and Alexa Fluor 594-conjugated secondary antibodies (Abcam) were loaded at room temperature for 2 h. The nuclei were stained with DAPI (Beyotime). Visualization of the images was through the BX63 microscope (Olympus) or LSM-980 confocal microscope (Carl Zeiss, Oberkochen, Germany).

### Statistical analysis

All the data are expressed as mean ± standard deviation (SD) from at least three experimental repeats. For the analysis of two groups, the unpaired Student's t-test was employed for normally distributed data, while the Mann–Whitney U-test was applied for non-normally distributed data. To assess the significance of differences in multiple comparisons, a one-way analysis of variance (ANOVA) with Tukey’s post hoc test was employed. All data were processed using SPSS (v.21.0) software (IBM SPSS Inc., Chicago, IL, USA), where a value of P < 0.05 was considered as statistically significant.

### Supplementary Information

**Additional file 1: Figure S1.** The gene expression of Rac1 in siRNA-transfected fibroblasts (A) and HaCaT cells (B) were evaluated by RT-qPCR analysis after treated with or without hPTH(1–34).

**Additional file 2: Table S1.** Primer sequences used in present study.

## Data Availability

The data generated or analyzed during this study are contained in this published article.

## Abbreviations

hPTH(1–34): Human Parathyroid Hormone (1–34)
Rac1: Ras-related C3 botulinum toxin substrate 1
HaCaT cells: Human immortalized epidermal cells
siRNA: Small interfering RNA
ECM: Extracellular matrix
DMEM: Dulbecco’s modified Eagle’s medium
FBS: Fetal bovine serum
CCK-8: Cell counting kit-8
IHC: Immunohistochemistry
PTHrP: Parathyroid hormone related protein
