# Supplementary figures and images for: Human Parathyroid Hormone (1–34) accelerates skin wound healing through inducing cell migration via up-regulating the expression of Rac1
Source: Cell Div. 2024 Feb 12;19:4. doi: 10.1186/s13008-024-00111-3 (PMC10860314; doi:10.1186/s13008-024-00111-3)

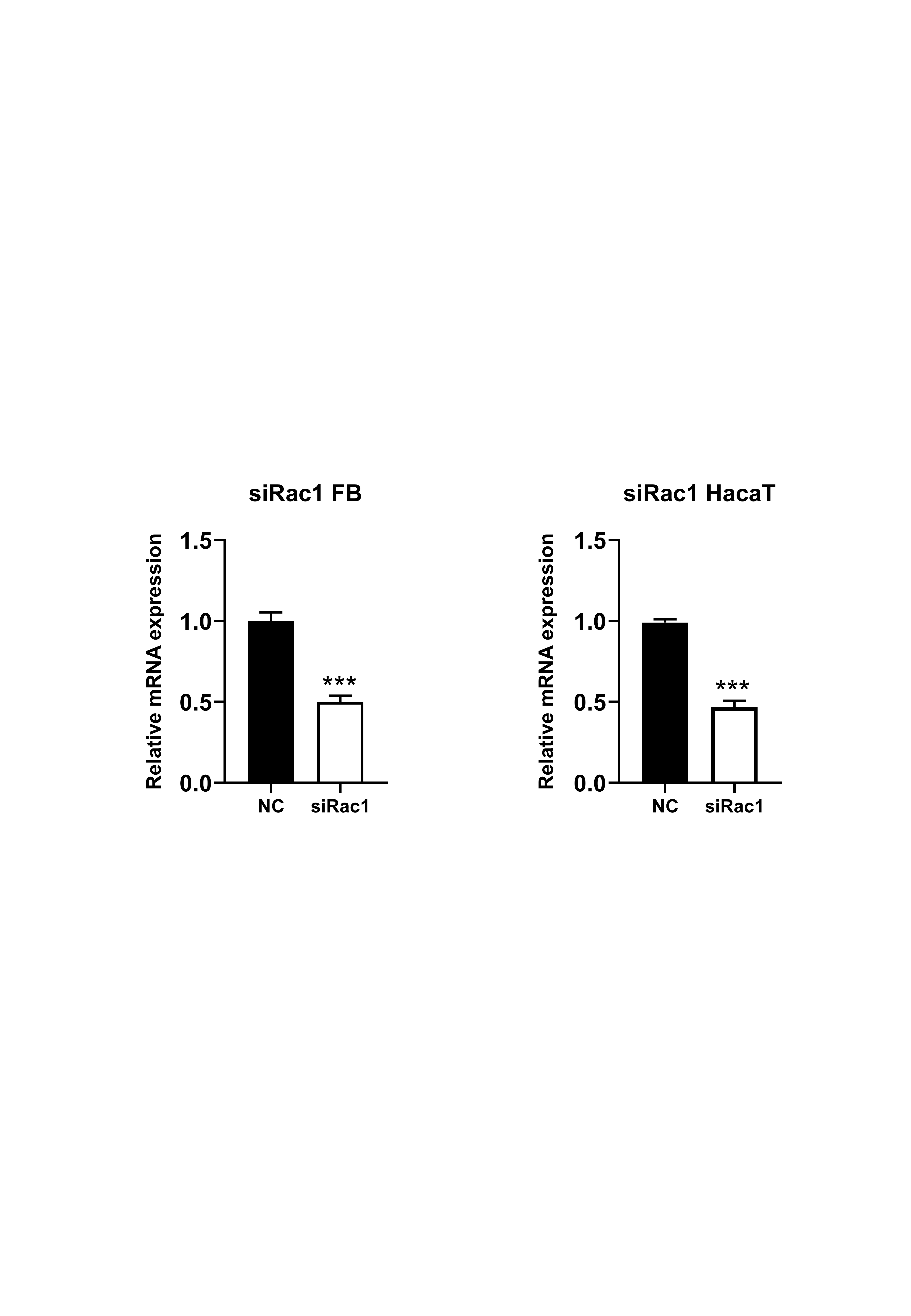

Supplement: Supplementary file 1 — Additional file 1: Figure S1. The gene expression of Rac1 in siRNA-transfected fibroblasts (A) and HaCaT cells (B) were evaluated by RT-qPCR analysis after treated with or without hPTH(1–34). [file 13008_2024_111_MOESM1_ESM.tif]
