# Supplementary material for: Human Parathyroid Hormone (1–34) accelerates skin wound healing through inducing cell migration via up-regulating the expression of Rac1
Source: Cell Div. 2024 Feb 12;19:4. doi: 10.1186/s13008-024-00111-3 (PMC10860314; doi:10.1186/s13008-024-00111-3)
Supplement: Supplementary file 2 — Additional file 2: Table S1. Primer sequences used in present study. [file 13008_2024_111_MOESM2_ESM.docx]

**Additional file Table 1.** Primer sequences used in present study.

| Gene | Forward (5’–3’) | Reverse (5’–3’) |
| --- | --- | --- |
| Rat Rac1 | GGCGAAAGAGATCGGTGCT | AAGCGTACAAAGGCTCCAGG |
| Rat GAPDH | ATGGCTACAGCAACAGGGT | TTATGGGGTCTGGGATGG |
| Human Rac1 | ATGTCCGTGCAAAGTGGTATC | CTCGGATCGCTTCGTCAAACA |
| Human GAPDH | ATCCCATCACCATCTTCC | GAGTCCTTCCACGATACCA |
